# Targeting NK Cell Inhibitory Receptors for Precision Multiple Myeloma Immunotherapy

**DOI:** 10.3389/fimmu.2020.575609

**Published:** 2020-11-12

**Authors:** Helmi Alfarra, Jackson Weir, Stacy Grieve, Tony Reiman

**Affiliations:** ^1^ Department of Biology, University of New Brunswick, Saint John, NB, Canada; ^2^ Department of Oncology, Saint John Regional Hospital, Saint John, NB, Canada; ^3^ Department of Medicine, Dalhousie University, Saint John, NB, Canada

**Keywords:** natural killer cell, immune checkpoint inhibitor, inhibitory receptors of lymphocytes, multiple myeloma, immunotherapy, precision medicine, chimeric antigen receptor NK, monoclonal antibody therapy

## Abstract

Innate immune surveillance of cancer involves multiple types of immune cells including the innate lymphoid cells (ILCs). Natural killer (NK) cells are considered the most active ILC subset for tumor elimination because of their ability to target infected and malignant cells without prior sensitization. NK cells are equipped with an array of activating and inhibitory receptors (IRs); hence NK cell activity is controlled by balanced signals between the activating and IRs. Multiple myeloma (MM) is a hematological malignancy that is known for its altered immune landscape. Despite improvements in therapeutic options for MM, this disease remains incurable. An emerging trend to improve clinical outcomes in MM involves harnessing the inherent ability of NK cells to kill malignant cells by recruiting NK cells and enhancing their cytotoxicity toward the malignant MM cells. Following the clinical success of blocking T cell IRs in multiple cancers, targeting NK cell IRs is drawing increasing attention. Relevant NK cell IRs that are attractive candidates for checkpoint blockades include KIRs, NKG2A, LAG-3, TIGIT, PD-1, and TIM-3 receptors. Investigating these NK cell IRs as pathogenic agents and therapeutic targets could lead to promising applications in MM therapy. This review describes the critical role of enhancing NK cell activity in MM and discusses the potential of blocking NK cell IRs as a future MM therapy.

## Introduction

Multiple myeloma (MM) is characterized by the accumulation of malignant plasma cells (PCs), resulting in increased monoclonal protein in the blood and urine (1). MM represents 1% of cancers and 13% of hematological malignancies, with a higher prevalence in aging populations (2, 3). In 2019, approximately 3,300 Canadians were newly diagnosed with MM, and 1,550 Canadians died from this disease (4). MM is a progressive disease that begins as an asymptomatic precursor called monoclonal gammopathy of undetermined significance (MGUS), before developing into smoldering MM (SMM), and ultimately, fully active symptomatic MM.

In recent years, there have been several notable therapeutic advancements for MM. Hematopoietic stem cell transplantation (HSCT) (5), proteasome inhibitors (PIs), immunomodulatory drugs (IMiDs) (6), histone deacetylase inhibitors (HDACi) (7), and novel combinations therapies (8, 9) have significantly improved the control of MM and extended overall survival (OS) (2, 5, 7, 10, 11). However, MM remains incurable as most patients eventually relapse due to the development of resistance to these conventional treatments (12). Inherent intra- and inter-patient heterogeneity contributes to the lack of curative success for this disease. Additionally, MM is considered a disease of the immune system. Gradual immune dysregulation and impairment of NK cells, T cells, B cells, and dendritic cells (DCs) allow malignant plasma cells to escape immunosurveillance (2). A better understanding of the immune environment of MM may lead to alternative therapeutic strategies that re-engage the immune system to inhibit MM growth.

Natural killer (NK) cells are an intriguing immune cell type in MM given the recent development of monoclonal antibodies (mAbs), elotuzumab (anti-SLAMF7), and daratumumab (anti-CD38) that enhance NK cell-mediated tumour cell toxicity by activating the antibody dependent cellular cytotoxicity (ADCC) mechanism (13–15). Although these mAbs have improved the clinical outcomes of both newly diagnosed and relapsed or refractory MM (RRMM) patients, only a subgroup of patients responds to these mAbs, highlighting the complexity of MM. CAR-NK cell therapies and combinations of existing treatments also work to restore the innate killing capacity of NK cells in MM.

**Figure 1:**
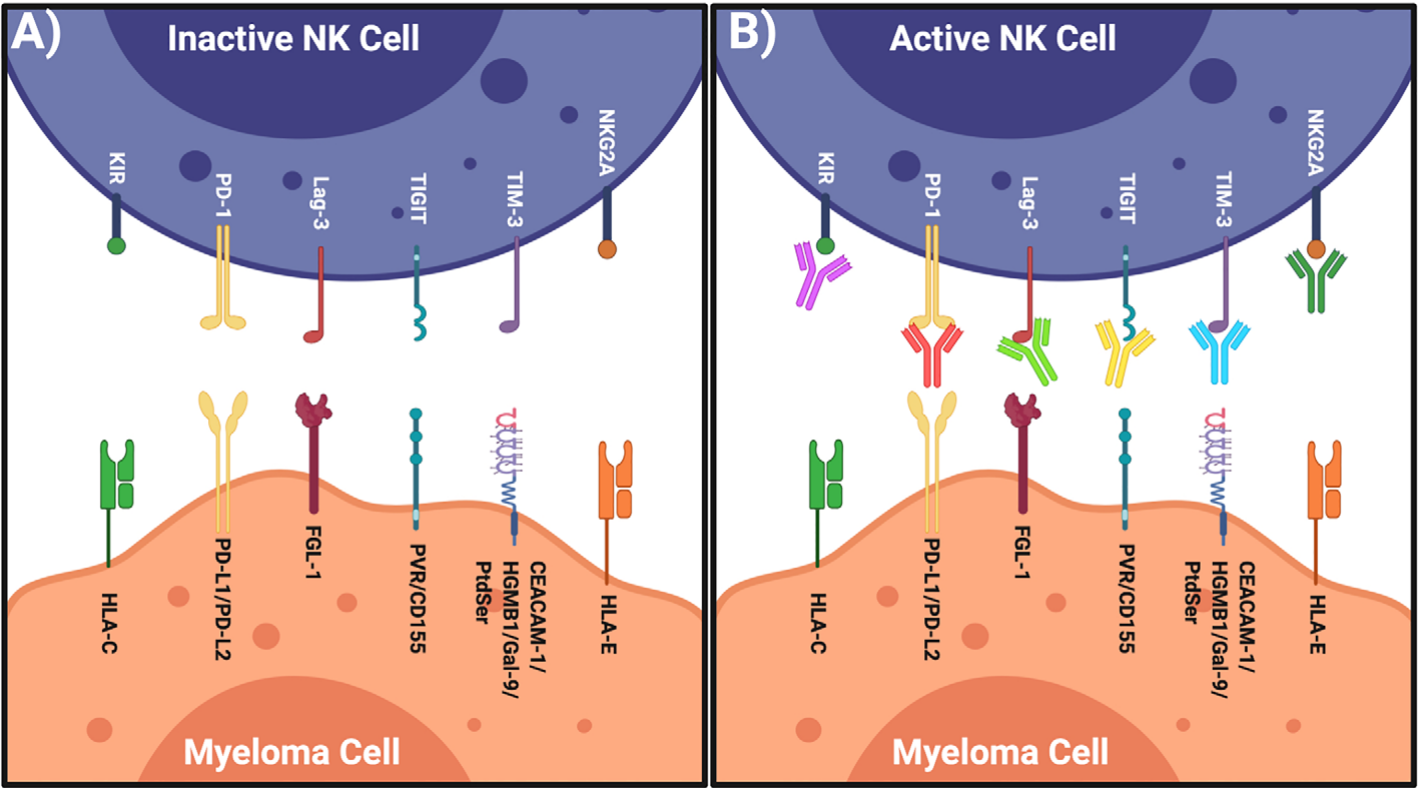
Restoring NK Cells by Targeting Their IRs. **(A)** Left: inactive NK cell has inhibitory receptors and complimentary ligands on the myeloma cell. **(B)** Right: NK cell activated when inhibitory axis is blocked *via* specific blocking mAbs. Figure created with BioRender.com.

Given the success of blocking T cell IRs in multiple cancer types, blocking the IRs on NK cells offers another possibility to enhance anti-myeloma cell immunity. This review discusses NK cell IRs (**Figure 1**) and their potential as novel NK cell-based MM immunotherapies to complement current treatment options. This line of investigation has the potential to maximize clinical benefit, thereby leading to efficient and safe immunotherapy options for MM patients.

## NK Cell Biology

NK cells are a cytotoxic subset of innate lymphoid cells (ILCs). They are the first responders against malignant and infected cells and are functionally classified by their innate capacity to eliminate cells without prior sensitization or recognition of presented antigens (16, 17). NK cells also produce cytokines and chemokines that stimulate other branches of the immune response including DCs and T cells (18, 19). Consequently, NK cells can limit cancer cell progression (20).

NK cells comprise 5% to 15% of peripheral blood lymphocytes (21, 22). Generally, they are defined as CD56^+ve^CD3^−ve^ and classified into two major populations—CD56^dim^ and CD56^bright^. The CD56^dim^ cells are considered the cytotoxic population and express more immunoglobulin-like receptors to detect stressed cells and induce cell death. CD56^bright^ cells are known as the pro-inflammatory cytokine releasers and specialize in promoting other components of the immune system through IFN-γ and TNF-α production (23–26). Notably, CD56^bright^ NK cells have been shown to display cytotoxic activity when primed with IL-15 (27).

**Figure 2:**
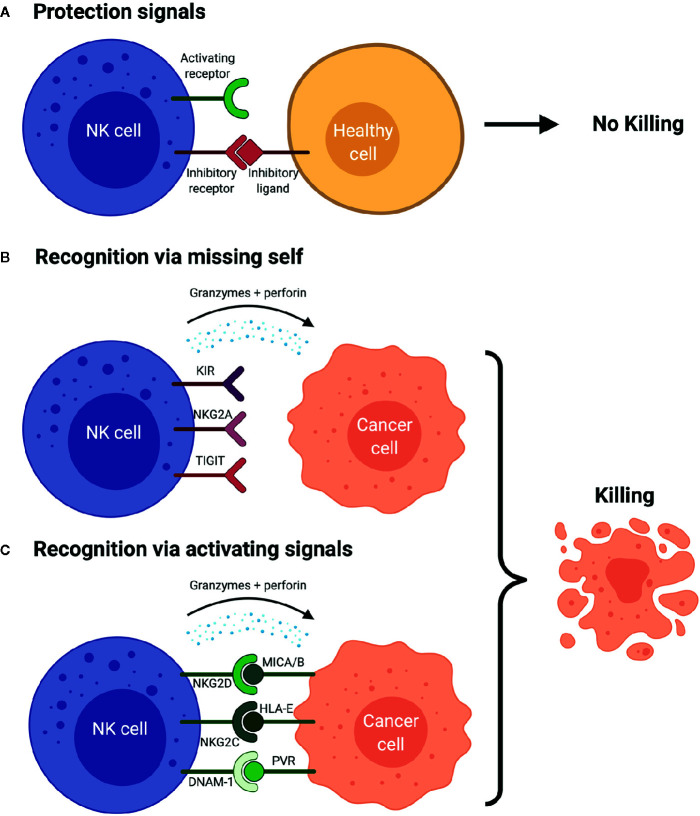
NK cell Surveillance of Cancer Cell **(A)** The presence of inhibitory signals and lack of activating signals prevents the activation of the NK cells which avoids the lysis of the healthy cells. **(B)** NK cell recognizes the cancer cell due to the lack of human leukocyte antigens (HLAs) and/or other inhibitory ligands on cancer cell (“missing-self hypothesis”), which results in production of cytokines, granzyme B and perforins that leads to the cancer cell killing. This scenario is simplified. Activation signals are still necessary to induce activation as the absence of inhibitory signals alone is usually insufficient. **(C)** NK cell is activated *via* the activating signals and the engagement with the activating ligands on the cancer cell in the lack of inhibitory signals, which leads to the production of perforins and granzyme B and cytokines, which ultimately yields cancer cell killing. Figure created with BioRender.com.

When an NK cell encounters a cell, it does not necessarily induce cell lysis. Instead, cytotoxicity is dependent on expression of AR and IRs on the NK cells that are engaged by specific ligands expressed on target cells (28). For example, inhibitory receptors expressed on the surface of a NK cell bind inhibitory ligands on a healthy cell (29). Without any activating ligands on the healthy cell’s surface, the inhibitory signal predominates and there is no cell lysis (**Figure 2A**). The inhibitory ligands human leukocyte antigen class I (HLA-I) are expressed on most healthy cells, preventing NK-mediated cell lysis. The first-described mechanism of NK cell function the “missing-self hypothesis” showed that when target cells lacked expression of this “self” ligand, HLA-I, the effector NK cells were free to become activated and remove the target cells (17) (**Figure 2B**). Interestingly, cancer cells often downregulate HLA-I (30), but we now know the story is much more complex and includes many additional IRs and ligands (**Figure 1**).

While the “missing self” mechanism of cell death works primarily through the lack of inhibitory signals, NK cells can also kill cancer cells with adequate activation signals (**Figure 2C**). For example, natural killer group 2D (NKG2D) is an activating receptor which recognizes HLA-I polypeptide-related sequence A/B (MICA/B), and UL16 binding proteins 1–6 (ULBP1-6) activating ligands. NKG2D ligands (NKG2DL) are often upregulated on malignantly transformed cells for NK cell detection (28, 31). NK cells express other ARs and a detailed review of their function can be found elsewhere (32, 33).

When an NK cell comes in contact with a stressed cell, different patterns of inhibitory and activating ligand expression are detected through the NK cell’s IRs and ARs and the balance of these ligands and receptors dictates NK cell function. Activated NK cells can send suicide or self-destruction signals to the target cell and induce cell lysis through direct exocytosis of granzyme and perforin (34–36). Stimulated NK cells have also been shown to kill cancer cells *via* apoptotic pathways (i.e., Fas or TRAIL) and cytokine production (i.e., TNF-α and IFN-γ) that are important for both innate and adaptive immune responses (36).

NK cells are integral members of anti-cancer immunity. While the cytotoxic mechanisms presented above represent ideal scenarios, the complex cancer immune microenvironment is marked by NK cell dysfunction and impairment. Deciphering how NK cell dysfunction contributes to tumorigenesis is essential to improve patient outcomes.

## NK Cell Surveillance of Malignant Cells

Several studies have reported the importance of NK cells in the immunosurveillance of tumor growth. An epidemiological study described that low activity of NK cells increased the risk of cancer specifically stomach, lung, and intestine (37). Other studies in mice models and humans associated tumor relapse and metastasis with decreased NK cell immunosurveillance (38–41). Preclinical studies are consistent with clinical data demonstrating that an NK cell-mediated immune response affects tumor formation and metastases (40, 42, 43). It has also been reported that the infiltration of NK cells into some solid tumors affects tumorigenesis in these cancer types (44–49) and can serve as a positive prognostic factor (48, 50).

### NK Cells in Hematological Cancers

NK cell numbers and functions have been linked to the prognosis of different blood cancers (41, 51–55). For example, the presence of NK cells in the bone marrow (BM) of Acute Lymphoblastic Leukemia (ALL) at diagnosis correlated with improved response to chemotherapy combinations treatments and increased tumor remission rates (55). Additionally, the predominance of activated NK cells expressing NKp46, FasL and KIR2DL5A in ALL patients was associated with better leukemia control after treatment with methotrexate, cytarabine, and hydrocortisone (54). Similarly, IFN-γ release by NK cells, an indication of their active state, was a favorable prognostic marker in chronic myeloid leukemia (CML) (56). Finally, a lower percentage of NK cells in the peripheral blood have been associated with a poorer prognosis of pediatric non-Hodgkin’s lymphomas (NHL), adult chronic lymphocytic leukemia (CLL), diffuse large B-cell lymphoma (DLBCL) (41, 51, 52), and high-risk myelodysplastic syndrome (MDS) (57). Despite these studies supporting an impact of NK cells on disease progression, the relationship between NK cells and outcome in the setting of some cancers is still controversial. It remains to be confirmed whether the increase in NK cell number and activity is a result of tumor progression or an indication of antitumor immune response.

Likewise, the correlation between NK cells and the progression of MM is controversial (58–65). Some studies have shown a decrease in NK cell number in the peripheral blood of myeloma patients when compared to healthy controls (58–60), while other studies demonstrated an increase or no difference (61–63). A recent study using single-cell RNA sequencing showed enrichment of NK cell populations during MGUS, and phenotypic shifts later in MM progression that potentially point to a compromised immune system (66). Discrepancy is also observed in terms of NK cell functionality where either reduced NK cell function (65) or high NK cell function (59, 67) are linked with advanced clinical stage, high-risk disease and reduced survival.

Due to the complexity, heterogeneity, and plasticity of NK cells in cancer patients, the discrepancies are difficult to distill into a single explanation (67). One likely explanation seems to relate back to which NK cell subpopulations are being measured. For example, it was reported that MM patients with a high CD56^+ve^CD3^-ve^ subset had a poorer prognosis. By contrast, patients with high of CD57^+ve^CD8^-ve^ subset of NK cells had a better prognosis (59). This suggests that there are essential distinctions to be made between these two populations, and that the existence of mature NK cells (CD57^+ve^) in early stage patients, but not the immature subset, forecasts good outcomes (59). Secondly, the production of NK cell stimulatory cytokines (e.g., IL-7 and IL-12) by myeloma cells in some patients could also explain differences in NK cell activity or number observed in different patient subsets since some patients’ immune system may be trying to control the disease (59, 68).

**Figure 3:**
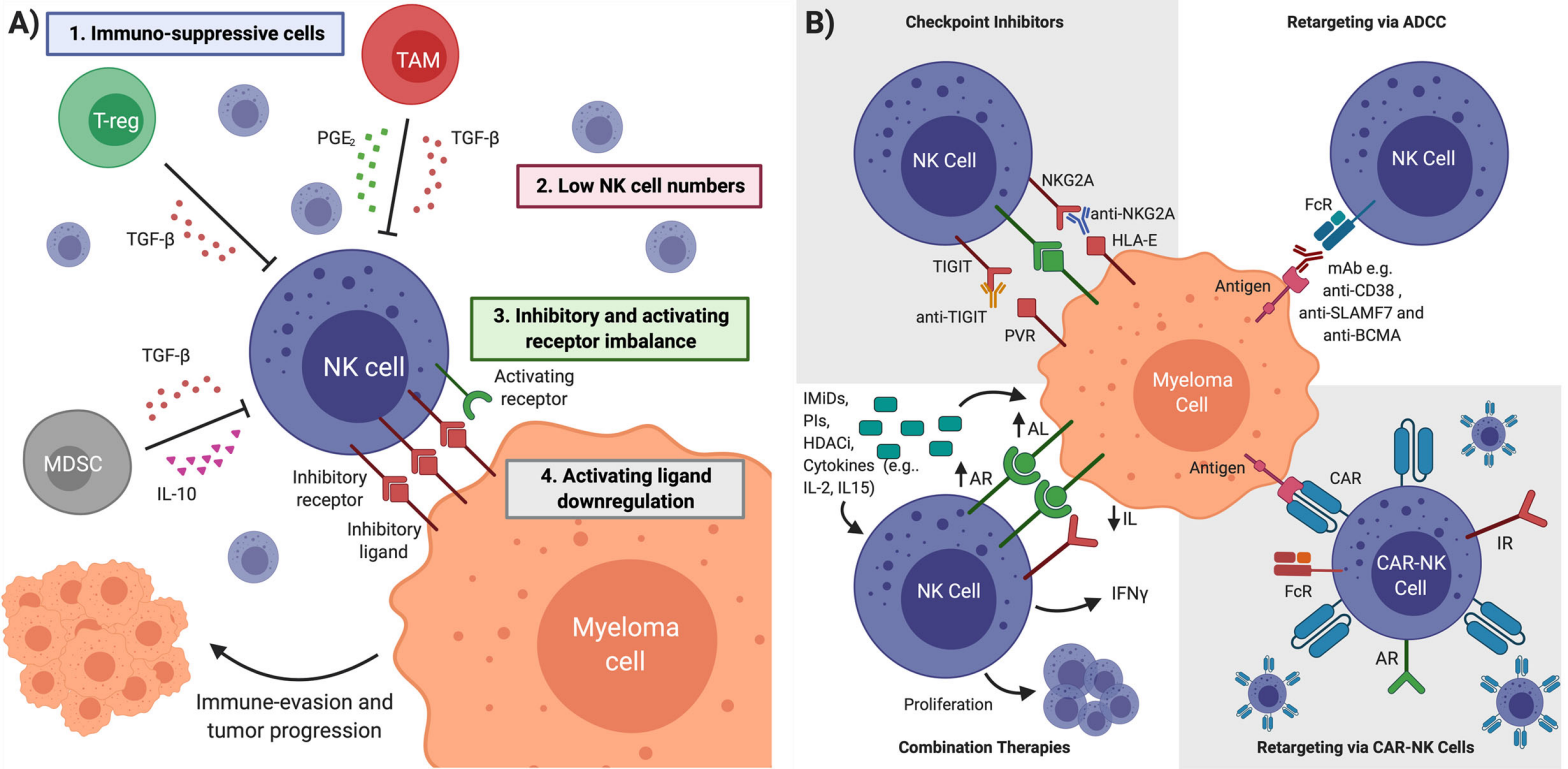
NK Cell Restoration Approaches for Multiple Myeloma Immunotherapy. **(A)** NK cell impairment in MM is characterized by (1) immunosuppressive cells and cytokines (2) low NK cell numbers (3) inhibitory and activating receptor imbalance in favor of NK cell inhibition (4) downregulation of activating ligands on cancer cell. Multiple myeloma cells in an impaired NK cell environment evade detection and continue proliferation. **(B)** Several therapeutic interventions can overcome NK cell impairment. Checkpoint inhibitors block inhibitory receptors to unleash NK cell cytotoxicity. Antibody-dependent cellular cytotoxicity (ADCC) uses mAbs designed to bind tumor-specific antigens and mediate anti-myeloma NK cell killing. Immunomodulatory drugs (IMiDs), proteosome inhibitors (PIs), histone deacetylase inhibitors (HADCi) and cytokines can upregulate activating ligands (ALs) and downregulate inhibitory ligands (ILs) on cancerous cells, upregulate activating receptors (ARs) and IFN-*γ* in NK cells, as well as promote NK cell proliferation. CAR-NK cells are engineered to target tumor-specific antigens and kill cancerous cells upon introduction to patient. TGFβ is Transforming growth factor beta, PGE2 is Prostaglandin E. Figure created with BioRender.com.

Finally, heterogeneity in ligand and receptor expressions within patient subsets may account for variability in research studies. For example, some patients may have reduced levels of activating ligands such as MICA/B that normally send signals to the activating receptor of the NK cell, NKG2D (**Figure 3A**). This reduction in MICA/B levels on the myeloma cells leads to loss of NK cell activation through NKG2D receptor, allowing MM cells to evade NK cell surveillance (69, 70). Alternatively, HLA-I ligand upregulation on MM cells may also block NK cell activity (67, 71). Interestingly, myeloma cells harvested from MM patients from the BM early in disease progression expressed a relatively low level of HLA Class I ligands and were subsequently responsive to the NK cell mediated cytotoxicity. As disease progressed into fully active myeloma, the tumorigenic MM cells displayed higher HLA-I ligands expression, rendering them more resistant to NK cell-mediated cell death (72). Another mechanism that may also be responsible for altered NK cell function in MM includes upregulation of IRs such as PD-1 on NK cells found in the peripheral blood or BM of MM, which may lead to decreased NK cell function due to its engagement with its ligands PD-L1/2 on MM cells (71, 73, 74) (**Figure 3A**).

These studies not only provide a possible explanation for a discrepancy regarding the role of NK cells in MM, but also point to how an imbalance in ARs and IRs could lead to NK cell dysfunction in MM (64, 75) (**Figure 3A**). Upregulation of various IRs on the surface of NK cells combined with the overexpression of their cognate ligands on the cancer cells can be a dynamic escape tactic used by cancer cells to hinder NK cell activity (**Figure 3A**). A better understanding of the interplay between MM cells and NK cells may lead to the rational development of novel NK cell-based therapies.

## Restoring NK Cells for MM Therapy

NK cells play an integral role in tumor surveillance, but are thought to be dysfunctional in MM patients. Immunosuppressive cells and cytokines, low NK cell numbers, IR and AR imbalance, and AR downregulation all lead to NK cell impairment and their inability to kill MM cells (**Figure 3A**). Given this suppressive environment, several therapeutic interventions have been established to restore anti-myeloma NK cell function. While this review focuses primarily on targeting NK IRs, there are several other clinically relevant treatment strategies that can re-engage NK cells to mediate an anti-MM phenotype.

### mAbs ADCC

The mAb-ADCC approach recruits NK cells to myeloma cells that may otherwise be unrecognizable as stressed cells due to low activating ligand expression and IR/AR imbalance. As CD56^bright^ NK cells mature to CD56^dim^ cells, they express the Fcγ receptor III (also called CD16) that is important for ADCC against mAb-coated cancer cells (23–26). Patients treated with mAbs that bind tumor specific antigens on MM cells allow NK cells to recognize the Fc region and induce ADCC toward the MM cell (76) (**Figure 3B**).

Currently approved mAbs targeting MM cells include elotuzumab and daratumumab, targeting SLAMF7 and CD38 respectively. Both mAbs enhance NK cell cytotoxicity *via* ADCC (77, 78). Elotuzumab has also been shown to enhance NK cells through a secondary, indirect mechanism (79). The success of these antibody-based therapies may reflect the dynamic expression of their receptor targets. For example, after initial treatment, expression of these receptors was found to be decreased (80, 81). Alternatively, since CD38 is broadly expressed on NK cells, treatment with anti-CD38 mAbs may lead to a substantial depletion of the NK cell population (74). As a whole, mAbs have been successful in treating a subset of MM patients.

### CAR-NK Cell Therapy

Adoptive NK cell therapy aims to restore patient innate immune surveillance and control cancer progression by supplementing with new NK cells. This approach has shown promise against MM and other hematological malignancies including leukemia (82). Chimeric antigen receptor NK (CAR-NK) cells are a type of adoptive transfer therapy that uses genetically manipulated NK cells to specifically target tumor antigens. CAR-NK cell development builds on the recent success of CAR-T cells in cancer therapy. The significant clinical outcomes of anti-CD19 CAR-T cells in MM justified the creation of CAR-T cells targeting other antigens expressed on myeloma cells, including CD38 (83), CD138 (84), SLAMF7 (85), SLAMF3 (86), CD56 (87), NKG2D (88), and most successfully BCMA (89). However, despite their early success, CAR-T cells are not exempt from limitations such as Graft-versus-Host disease (GvHD), cytokine release syndrome, neurotoxicity and off-tumor/on target toxicity (90, 91) that threaten patient safety. CAR-NK cells were developed to overcome some of the limitations of CAR-T cells. Compared to CAR-T cells, CAR-NK cells have shorter half-lives which may reduce some of the toxic side effects such as the induction of GvHD and the production of cytokines (92, 93). In addition, NK cells inherently express a range of ARs that prime them for activation (**Figure 3B**). NK cells also express Fc receptors that can enhance NK cell ADCC, suggesting that combination CAR-NK therapy with mAb therapy may be relevant therapeutic avenue to explore. Importantly, unlike CAR-T cells, CAR-NK cells are not HLA restricted; therefore, sources of CAR-NKs could include primary NK cells, NK cell lines (e.g., NK-92), umbilical cord blood or induced pluripotent stem cells (iPSCs) (94, 95). Taken together, these properties suggest CAR-NK cells could be a favourable alternative to CAR-T cells. Interestingly, NK cell lines make great candidates for NK genetic engineering allowing for the production of an “off-the-shelf therapeutic”. CAR-NK cells targeting several different antigens, including CD138, SLAMF7, CD19, CD20, CD33 and CD123, using both primary NK cells and NK cell lines, are currently being investigated in pre-clinical studies of both solid and hematological malignancies (96, 97). In hematological malignancies, anti-CD19 CAR-NK-92 cells improved cytotoxicity against leukemia cells and non-Hodgkin’s lymphoma or chronic lymphocytic leukemia (CLL) expressing CD19 (98, 99). In MM, preclinical results have shown efficacy of CAR-NK cells targeting CD138 (100). CAR-NK cells targeting BCMA, NKG2D (101), or SLAMF7 (102) are also being explored within a MM context.

Early clinical trials using anti-CD33 CAR-NK-92 cells showed no major adverse effects in relapsed/refractory acute myeloid leukemia (AML) patients, supporting the notion that CAR-NK cells could be a safe alternative to CAR-T cells in hematological malignancies (103). The first CAR-NK cell clinical trial in MM (NCT03940833) plans to use anti-BCMA CAR-NK cells to treat 20 patients with relapsed and refractory MM. Although still in the early stages, CAR-NK cells are becoming a promising NK cell-based therapy for overcoming the immunosuppressive environment of MM.

### Combination Therapies

IMiDs have become a staple of MM treatment in the last two decades. Although their canonical mechanism of action is not often thought to include NK cells, IMiDs can act to restore NK cell activity. IMiDs reduce the NK cell activation threshold (104), increase NK cell proliferation and enhance NK cell mediated cytotoxicity (105). Multiple studies also show lenalidomide or the chemotherapeutic melphalan increases activating ligand expression on MM cells (106, 107). Furthermore, *in-vitro* and *in-vivo* studies have shown that the combination of elutuzumab and lenalidomide enhanced anti-proliferative effects more than any single agent and was associated with increase NK cell activation as demonstrated by the stimulation of activating cytokine production and induction of MM cell death in *in vitro* co-culture assays. Interestingly, in *in-vivo* established MM xenografts, although NK cells recruitment to tumor sites was not associated with lenalidomide, this recruitment could be enhanced by the addition of elotuzumab, likely through an ADCC-mediated mechanism (108). Another *in-vitro* study on BM mononuclear cells from MM patients have been shown that combination of daratumumab-IPH2102 anti-killer cell immunoglobulin-like receptors (anti-KIR) with lenalidomide have improved the NK cell ADCC activity and myeloma cell lysis (108, 109) (**Figure 3B**).

Proteosome inhibitors, such as bortezomib, also enhance anti-MM NK cell killing by downregulating HLA-I (110), upregulating NKG2D and DNAM-1 ligands (106), and increasing tumor cell susceptibility to NK cell activity *via* upregulation of the TRAIL and FasL apoptotic pathways (111). Bortezomib in combination with elozuzumab and daratumumab have proven effective (112, 113). Histone deacetylase (HDAC) inhibitors might also upregulate activating ligand MICA and improve the anti-MM NK cell response (114).

Expectedly, certain cytokines have been shown to augment NK cell function in MM and other hematological cancers. These cytokines include IL-2 and TNF-α (114), IL-15 and IL-21 (115, 116). However, given the ability of different cytokines to upregulate other parts of the immune system at a considerably high level, there are significant risks associated with their use. Although IL-2, for example, has been approved for metastatic renal cell carcinoma and metastatic melanoma, it is not a standard treatment in monotherapy due to severe side effects in high doses (117). Moving forward, incorporating lose dose cytokines in combination with other treatments should be the focus.

## NK Cell IRs

Targeting NK IRs may unleash the breaks preventing NK cells from detecting and killing myeloma cells. Common IRs include KIRs, NK group 2 member A (NKG2A), T-cell immunoglobulin and mucin-domain containing-3 (TIM-3), T-cell Ig and ITIM domain (TIGIT), V-domain Ig-containing suppressor of T cell activation (VISTA), programmed death-1 (PD-1), cytotoxic T-lymphocyte-associated protein 4 (CTLA4) and lymphocytic-activation gene 3 (LAG-3) (**Figure 1**). In order to overcome IR/AR imbalance and the altered activation threshold following AR downregulation, the use of immune checkpoint inhibitors to block IRs on NK cells will reduce the inhibitory signal thereby enhancing NK cell activation (**Figure 1**).

**KIRs** are a group of inhibitory and activating type I transmembrane glycoproteins expressed on most NK cells and some T-cell subsets (118, 119). Belonging to the immunoglobulin superfamily, KIRs have a transmembrane domain, a cytoplasmic tail, and two or three Ig-domains. Generally, short cytoplasmic domains (KIR2DS/KIR3DS), transduce activating signals to the lymphocyte while long cytoplasmic domains (KIR2DL/KIR3DL) inhibit lymphocyte-mediated cytotoxicity (120).

The significant ligands of the KIR family include the HLA-I molecules. The most well-characterized inhibitory ligand is HLA-C where KIR2DL1 binds the C2 allele of HLA-C, and KIR2DL2 binds the C1 allele (121, 122), although activating KIRs have also been shown to interact with HLA-C (123). Inhibitory KIRs also interact with HLA-B (124), HLA-A (125), and HLA-F (126). Although KIR expression on NK cells was initially thought to vary stochastically, it is now understood that NK cells undergo an educational process as they mature, altering the expression of specific KIRs to maximize the balance between self-tolerance and effective defense (127). Additionally, a study evaluating hematopoietic stem cell transplantation in leukemia patients showed that NK cells expressing KIR2DL2/3 inhibitory receptors were still able to kill HLA-C expressing cancer cells if KIR2DS1 activating receptor was co-expressed (128), suggesting that KIR activating receptor profiles of patients should also be considered when targeting the KIR-HLA-I blockade.

In non-transplantation settings, blocking the KIR-ligand axis may improve tumor immunity, similar to other checkpoint inhibitors (129). Thus, several researchers have started developing anti-KIR antibodies to effectively create missing-self tumor cells and to lower the NK cell activation threshold. Pre-clinical studies showed that the anti-KIR mAb 1-7F9 (also called IPH2101) blocked inhibitory receptors KIR2DL1/2/3 and activated antitumor NK cytotoxicity against HLA-C expressing AML cells (130). In MM, combining lenalidomide with IPH2101 in mouse models augmented the anti-myeloma NK cell response and increased tumor clearance (131).

Despite this pre-clinical success, heterogeneity in KIR expression can make mAbs targeting of these KIRs difficult in a clinical setting (132). A phase I trial (NCT00552396) (n=32) investigated IPH2101 as monotherapy in MM patients and found increased NK cell cytotoxicity against MM cells *ex-vivo*. IPH2101 appeared safe and tolerable at the dose that achieved full inhibitory KIR saturation (133). Another phase I trial (NCT01217203) (n=15) by the same research group this time they investigated the IPH2101-Len combination. Several patients experience severe adverse events, and five reported objective responses (134).

A phase II clinical trial treating MM patients with anti-KIR2D mAb (IPH2101) showed a surprising decrease in NK cell activity and KIR2D expression (129) that is thought to be driven by monocyte trogocytosis, a process of surface protein exchange at the immunological synapse. The same group ran another phase II trial (NCT01248455) (n=9) studying IPH2101 as monotherapy in SMM patients. They postulated treating SMM rather than later stage MM could be the ideal time point for NK cell-based therapy to prevent the more aggressive MM cells from mediating an anti-immune response. IPH2101 was well tolerated with no grade 3 or 4 toxicities, however the study was discontinued due to a lack of patients meeting the defined primary objective (50% decline in M-protein) (135). Due to limited success in these early clinical studies, targeting KIRs may be more effective in combination with other therapies that augment KIR immunogenetics and education of NK cells (136, 137). So far, seven anti-KIR mAb clinical trials in MM are in progress (**Table 1**). One of the ongoing trials is evaluating anti-KIR mAbs in combination with anti-PD1 therapies, anti-CTLA-4 or daratumumab in myeloma and lymphoma patients (NCT01592370). Several anti-KIR combination therapy clinical trials are also in the recruitment stages. Combinations with other drugs or interventions such as CAR-T cell therapy, CAR-NK cell therapy, or in the setting of adoptive cellular transfer therapy may also improve the response. Trials with greater patient enrollment and the ability to characterize individual patient NK cell profiles would offer value and help identify patients profiles that respond best to this intervention.

**Table 1:** Selected clinical trials evaluating the safety, tolerability and efficacy of potential NK IRs for Multiple Myeloma NK cell-based immunotherapy (access date: August 10, 2020).

Receptor	Trial	Disease	Drugs	Phase	Participants	Results	Last Update Posted
KIR	NCT01217203	Relapsed multiple myeloma	IPH2101, Lenalidomide	I	15	Complete; Objective response in 5 patients; Severe adverse events in 5 patients; No autoimmunity	February 28, 2014
	NCT01222286	Smoldering multiple myeloma	IPH2101	II	30	Complete; No objective response; Adverse events in all patients	May 9, 2014
	NCT00999830	Multiple myeloma	IPH2101	II	27	Completed; Primary response in one patient (based on M-protein); Adverse events in 25 of 27 patients	March 24, 2016
	NCT00552396	Multiple myeloma	Anti-KIR (1-7F9)	I	32	Complete; No dose-limiting toxicity; Severe adverse event in 1 patient; Increased patient NK cell cytotoxicity against MM *ex vivo*	March 31, 2016
	NCT02252263	Multiple myeloma	Elotuzumab, Lirilumab, Urelumab	I	44	Complete; No results	November 1, 2017
	NCT01592370	Non-Hodgkin’s lymphoma, Hodgkin lymphoma, multiple myeloma	Nivolumab, Ipilimumab, Lirilumab, Daratumumab, Pomalidomide, Dexamethasone	I/II	375	Ongoing	May 18, 2020
	NCT01248455	Multiple myeloma, smoldering multiple myeloma	Anti-KIR	II	9	Terminated; Lack of patients meeting primary objective (50% decline in M-protein)	November 19, 2019
NKG2A	NCT02921685	Hematologic malignancies	Monalizumab	I	18	Ongoing	September 19, 2018
LAG-3/TIGIT	NCT04150965	Relapsed refractory multiple myeloma	Elotuzumab, Pomalidomide, Dexamethasone, Anti-LAG-3, Anti-TIGIT	I/II	104	Ongoing	July 7, 2020
PD1	NCT02903381	Smoldering multiple myeloma	Nivolumab, Lenalidomide, Dexamethasone	II	41	Suspended; Safety concerns	July 21, 2020
	NCT02636010	Multiple myeloma	Pembrolizumab	II	20	Complete; No results	April 29, 2020
	NCT02331368	Multiple myeloma	Autologous Stem Cell Transplant, Melphalan, Lenalidomide, MK-3475	II	32	Terminated; Complete response in 7 of 23 evaluable patients; Severe adverse events in 14 of 32 total patients	July 27, 2018
	NCT03848845	Multiple myeloma	GSK2857916, Pembrolizumab	II	40	Ongoing	August 5, 2020
	NCT03605719	Recurrent plasma cell myeloma	Carfilzomib, Dexamethasone, Nivolumab, Pelareorep	I	62	Ongoing	November 25, 2019
	NCT03530683	Lymphoma, multiple myeloma	TTI-622, Rituximab, PD-1 Inhibitor, Proteasome-inhibitors	I	156	Ongoing	September 12, 2019
	NCT03111992	Multiple myeloma	PDR001, CJM112, LCL161	I	26	Complete; No results	April 21, 2020
	NCT03357952	Multiple myeloma	Daratumumab, JNJ-63723283	II/III	10	Ongoing; All patients with treatment emergent adverse events; No dose limiting toxicity so far	January 3, 2020
	NCT03221634	Multiple myeloma	Pembrolizumab, Daratumumab	II	0	Withdrawn; Business reasons	March 25, 2019
	NCT03292263	Multiple myeloma	Melphalan, Nivolumab, Autologous Stem Cell Transplantation	I/II	30	Ongoing	March 17, 2020
	NCT02906332	Multiple myeloma	Pembrolizumab, Lenalidomide, Dexamethasone	II	16	Ongoing; Combination is well tolerated; Preliminary data show potential efficacy	January 31, 2020
	NCT02807454	Multiple myeloma	Daratumumab, Durvalumab, Pomalidomide, Dexamethasone	II	37	Ongoing	July 2, 2020
	NCT02685826	Multiple myeloma	Durvalumab, Lenalidomide, Dexamethasone	I/II	56	Ongoing; Majority of patients with adverse events; Dose-limiting toxicity in 2 patients	April 27, 2020
	NCT02616640	Multiple myeloma	Durvalumab, Pomalidomide, Dexamethasone	I	114	Ongoing	April 17, 2020
	NCT02576977	Multiple myeloma	Pembrolizumab, Pomalidomide, Dexamethasone	III	251	Terminated; Anti-PD1 treatment combination had unfavourable benefit-risk profile in relapsed refractory multiple myeloma	July 31, 2020
	NCT02579863	Multiple myeloma	Pembrolizumab, Lenalidomide, Dexamethasone	III	310	Terminated; Anti-PD1 treatment combination had unfavourable benefit-risk profile in newly diagnosed multiple myeloma	August 3, 2020
	NCT02289222	Multiple myeloma	MK-3475, Pomalidomide, Dexamethasone	I/II	48	Terminated; Due to inclusion of an IMiD in combination with pembrolizumab	November 5, 2019
	NCT02077959	Multiple myeloma	Lenalidomide, Pidilizumab	I/II	20	Complete; No results	May 30, 2019
	NCT02036502	Multiple myeloma	Pembrolizumab, Lenalidomide, Dexamethasone, Carfilzomib	I	77	Complete; Tolerable safety profile; Notable anti-tumor activity	July 13, 2020
	NCT02726581	Multiple myeloma	Nivolumab, Elotuzumab, Pomalidomide, Dexamethasone	III	348	Ongoing	August 10, 2020

**NKG2A** is an inhibitory receptor that belongs to the C-type lectins. It is a type II membrane receptor that forms a heterodimer with CD94 (138). NKG2A/CD94 is primarily expressed on NK cells and some T cells (139). Nearly half of the circulating NK cells express NKG2A/CD94. While its expression corresponds with a lack of KIR expression (140), other IRs including PD-1 and LIR-1 can be co-expressed with NKG2A/CD94 (139–141). NKG2A/CD94 recognizes the HLA-E ligand, a non-classical HLA class I molecule. Typically, HLA-E is expressed by healthy cells. Therefore, its interaction with NKG2A/CD94 represses activation signals and reduces cytokine secretion and NK cell cytotoxicity (142–146). NKG2A competes with the activating receptors NKG2C and NKG2E for the binding to HLA-E (17, 147, 148).

Both ligand and receptor are highly expressed in patient samples across tumor types (149–153). Even though intra-tumoral NKG2A^+ve^ NK cells are seen in the tumor microenvironment, the upregulation of HLA-E by cancer cells implies that these NK cells are functionally exhausted. In this way, high expression of HLA-E or exhausted NKG2A^+ve^ NK cells are associated with poor prognosis in different cancers (143, 144, 154–158). These observations suggest that inhibiting NKG2A/CD94 is a possible strategy that will release NK cell activity through checkpoint blockade therapy.

As proof-of-principle that NKG2A is important for NK cell activity and that it might serve as a relevant therapeutic target, NKG2A protein expression was knocked out in a human retroviral NK cell model to generate NKG2A-null NK cells. When NKG2A expression was lost, these cells showed higher cytotoxicity toward HLA-E positive cancer cells (159). Hence, reducing NKG2A expression or inhibiting it may provide an effective treatment strategy alone or in and combination therapy (150).

Accordingly, monalizumab, a novel IgG4 humanized antibody developed to block CD94/NKG2A, was shown to cause cancer cell death (139). Although not studied in MM, preclinical and clinical investigations in different cancer settings provide evidence that blocking CD94/NKG2A is a viable therapeutic option. For example, *in-vitro* pre-clinical investigations using NK cells from chronic lymphoid leukemia (CLL) patients showed that monalizumab restored their cytotoxicity (143). Similarly, using cells from previously treated patients with head and neck squamous cell carcinoma, it was shown that monalizumab boosted NK ADCC as well as unleashed CD8+ T cells. Monalizumab also synergized with anti-PD-1/PD-L1 blockade and cetuximab mAb combined therapy in *in-vitro* assays (139). Further *in-vivo* mouse studies revealed that the anti-NKG2A antibody could kill engrafted primary human leukemia through an NK cell-mediated mechanism (158).

Although there is limited knowledge regarding the role of NKG2A in MM, *in-vitro* experiments showed that the level of HLA-E on the myeloma cells could potentiate the inhibition of NKG2A (67). This suggests that anti-NKG2A may be beneficial for patients that have functional levels of HLA-E (67, 148), although studies proving this hypothesis are still ongoing. This preliminary evidence, combined with positive preclinical studies in other malignancies (149, 156), points to the therapeutic potential of blocking NKG2A in MM. An ongoing phase I clinical trial (NCT02921685) is investigating monalizumab as monotherapy in patients with hematologic malignancies that were previously treated with an allogeneic HSCT (**Table 1**).

**TIM-3** is an IR expressed on functional and mature NK cells and other lymphocytes. TIM-3 interacts with specific ligands that include HMGB1 (high mobility group protein B1 proteins), CEACAM-1 (carcinoembryonic antigen cell adhesion molecule 1), PtdSer (phosphatidylserine), and galectin-9 (160–162). Engagement of TIM-3 with its ligands decreases cytokine production and NK cell toxicity, which eventually leads to tumor immunity and disease progression (163–165).

Like other IRs, expression of TIM-3 was also observed in circulating NK cells from cancers including lung adenocarcinoma (164), gastric cancer (166), advanced melanoma (167), bladder cancer (168), and follicular B-cell NHL. Upregulation of TIM-3 is linked to lymphocyte exhaustion and dysfunction (162, 169) and consequently can lead to poorer survival and tumor progression in several cancers (170).

Preclinical studies harvesting NK cells from patients with solid tumors have shown that blocking TIM-3 with anti-TIM-3 antibodies unleashed NK cell activity and induced IFN-γ production in NK cells (164, 171). Additional studies demonstrated that blocking TIM-3 reduced tumor growth in mouse models (169) or increased NK cell cytotoxicity against K562 leukemic cells (172).

It is important to note that there is still debate in the literature with contradictory assumptions about TIM-3 interaction with its ligands (173). For example, one study showed that binding of TIM-3 with Gal-9 stimulated IFN-γ release by NK cells, although this did not enhance NK cell-mediated toxicity (174). In another study, an anti-Gal-9 antibody that blocks its interaction with TIM-3 reduced IFN-γ production from NK cells from healthy donors when cocultured with primary AML blasts (160). Further, blocking of TIM-3 on IL15-stimulated NK cells showed little or no significant lysis of human pancreatic cancer cell lines (175). Similarly, and counter-intuitively, higher TIM-3 expression has been associated with increased tumor progression (166, 168, 171). Indeed, in a severe aplastic anemia mouse model, it was observed that the activity of the TIM-3^−ve^ NK cell population was higher than the TIM-3^+ve^ NK cell population (176). These results suggest that effect of TIM-3 blockade on NK cells may be tumor-specific and reflect the complex expression profiles of immune markers in both a cancer-specific and patient-specific manner.

TIM-3 blocking mAbs are under clinical investigation either alone or in combination with anti-PD-1/PD-L1 mAbs. Initial results of TIM-3 blocking in solid tumors reported a manageable safety profile and revealed early signs of activity even in patients previously treated with PD-1 or PD-L1 mAb (177, 178). Recent data from a current clinical trial on high-risk myelodysplastic syndrome (HR-MDS) and AML patients demonstrated that the combined treatment of the TIM-3 mAb MBG453 with decitabine was safe and well-tolerated and confirmed anti-tumor activity (179).

Overall, preliminary data suggests TIM-3 is a promising therapeutic target in several cancer types and supports the further clinical development of anti-TIM-3 inhibitors. No studies have specifically explored the role of TIM-3 in MM.

**TIGIT** is another inhibitory receptor expressed by both NK and T cells (180, 181). Several cancer types showed a high level of TIGIT on the tumor-infiltrating lymphocytes (TILs) (182). TIGIT recognizes the poliovirus receptor (PVR), also known as CD155 or Necl5, as well as the Nectin-2 (CD112), or Nectin-3 ligands that are overexpressed on multiple cancer types (183–185) and linked to unfavorable prognosis in several cancers (186, 187).

The majority of studies evaluating the role of TIGIT on tumor progression have focused on T cells and shown that TIGIT suppresses activity of T cells. In MM, refractory MM patients treated with DARA-pomalidomide combined therapy showed an increase in the exhausted T cells expressing CD28^–ve^LAG3^+ve^TIGIT^+ve^ (188, 189). Although NK cells were not evaluated, given that TIGIT is also expressed on NK cells, it is plausible that NK cells may also be inhibited.

When focusing on NK cells, TIGIT has been shown to both contribute to and inhibit tumor progression. In one study, Jia et al. (190) found that, although the number of TIGIT^+ve^ NK cells in AML patients were significantly lower in comparison to the healthy controls, these TIGIT^+ve^ NK cells also express high levels of ARs CD16 and CD160. Importantly, functional experiments showed an elevated expression of granzyme B and increased IFN-γ and TNF-α production by TIGIT^+ve^ NK cells compared with TIGIT^−ve^ NK cells. Therefore, the authors suggest that TIGIT expression on NK cells could be associated with activated and functional status of NK cell in AML and may impede tumor progression (190). The role of TIGIT is also complicated by the duality of the TIGIT ligand PVR. PVR is also a ligand recognized by the AR DNAM-1 that is expressed on NK cells. DNAM-1 has been shown to play a prominent role in NK cell-mediated anti-MM response (191). While TIGIT expression is increased in MM patients, DNAM-1 expression is decreased (192).

Given the dynamic nature of TIGIT expression on both NK cells and T cells as well as the dual role of TIGIT ligand PVR, particularly in MM, understanding how TIGIT affects NK cell function is critical. The complexity of this immune environment highlights the necessity to profile patients for expression of key IRs and ARs in order to better understand how to specifically harness NK cells to mediate an anti-tumor response in MM. CD8^+ve^T cells in the BM of newly diagnosed and relapsed MM patients expressed higher levels of TIGIT compared with those in the healthy group. In this cohort, the investigators observed moderate levels of TIGIT on the NK cells from newly diagnosed or relapsed patients (186). The same study investigated the anti-TIGIT mAb in an *in-vivo* model and showed that the anti-TIGIT mAb decreased myeloma disease burden in the BM and prolonged survival compared with control-Ig, or anti-PD-1 mAb-treated mice (186). This suggests that TIGIT expression is more dominant than PD-1 in the immunosuppressive function in MM, although the precise role of NK cells in this model was not examined.

The high expression of TIGIT on NK cells suggests that a blockade of TIGIT as a monotherapy or in combination with other therapies may reverse NK cell exhaustion and enhance their activation (181). There are numerous pre-clinical studies evaluating this hypothesis and examining how blocking TIGIT affects an anti-tumor immune response. In an *in-vivo* study, it was observed that TIGIT^−ve^ NK cells prevented colon tumor progression in mice. Similarly, the TIGIT blocking mAb overturned the exhaustion of antitumor NK cells, reactivating them and subsequently decreasing tumor growth (181). Importantly, it was observed that the absence of NK cells reduced the therapeutic effects of this TIGIT blocking mAb, suggesting that the presence of NK cells and the level of TIGIT expression on these NK cells may be critical for the clinical outcome of TIGIT blocking immunotherapy.

Blocking TIGIT in combination with other therapies has also shown pre-clinical success. Combined treatments with anti-TIGIT and anti-PD-1 antibodies in a mouse models showed significant growth reductions in lymphoma (193) and other tumors (194, 195) compared to monotherapies. Furthermore, co-expression of TIGIT with either PD-1 or TIM-3 has been correlated with a dysfunctional phenotype in TILs (195). Additional studies have shown that PVR expression can be induced by chemotherapy (192) or by IL-8 signaling through the CXCR1/CXCR2 axis (191), suggesting that a combination of anti-TIGIT mAbs with chemotherapy may be beneficial when patients’ immune cells have TIGIT expression.

Anti-TIGIT mAbs are now in phase I/II clinical trials as monotherapy or in combination with anti-PD-1 in solid tumors. Preliminary data from one trial (NCT02964013) showed a manageable safety profile and positive clinical response. Functional studies assessing how anti-TIGIT mAbs affect NK cells activity through cytokine production and NK cell degranulation in preclinical and clinical MM models may lead to an improved understanding of how to utilize NK cells in MM therapy.

**PD-1** is a surface receptor initially marked for its inhibitory function in T lymphocytes but is expressed on both T and NK cells (196). In healthy tissue, PD-1 regulates T cell activation and maintains self-tolerance (197, 198). PD-1 has two ligands (PD-Ls): PD-L1 and PD-L2 (199). When bound to either of these ligands, PD-1 inactivates its cognate NK or T cells. Since its discovery as an IR, PD-1 has become a central point of study for understanding negative immune cell regulation. Although the receptor plays a crucial role in protective immunity, PD-1 is of particular interest for its implications in tumor immune evasion. While PD-1 may protect healthy cells from immune cytotoxicity, some cancers harness PD-L1 as an evasion approach to bypass immune surveillance (200). While PD-L1 is lowly expressed in healthy human tissue, expression has been shown to be abundant across several different cancer types from different lineages (201). Expectedly, PD-L1 expression is upregulated in relapsed and refractory MM patients (202–205), although some studies report low expression of PD-L1 in MM (206, 207).

Recent work using mouse models argues PD-1 also plays a robust inhibitory role in NK cells, elucidating the responsiveness of anti-PD-1 treated patients with low tumor HLA expression who would not be expected to show high T cell activity (208). Therefore, studying PD-1 expression and function on NK cells may also be of therapeutic value.

While some studies show that PD-1 is only lowly expressed on activated NK cells (209) and that depletion of NK cells did not significantly affect therapeutic efficacy of the anti-PD-L1 therapy (210), some recent studies support the role of NK cells in the PD-1/PD-L1 inhibitory pathways (211). Some peripheral blood subpopulations display high levels of PD-1 expression and the interruption of the PD-1/PD-L1 axis could partially restore antitumor function of these NK cells in an ovarian cancer model (212). In MM, early preclinical studies of the interplay between PD-1 and PD-L1 in the NK cell showed that blocking PD-1 enhanced NK cell-mediated MM killing while preserving healthy cells (213). Additionally, recent findings showed that in MM patients samples, only NK cells that were positive for myeloma cell markers stained for PD-1, suggesting that PD-1 on NK cells was acquired from MM cells (214). As before, the discrepancy in research findings points to the complexity of the immune landscape and the need for a better understanding of the relationship between immune cell players and the expression of specific receptors and their ligands across patient datasets.

Despite success in solid tumors, anti PD-1/PD-L1 mAbs failed as monotherapy in MM (215) (**Table 1**). Failure as monotherapy has driven clinical testing of these antibodies in combination therapies. Data from phase I and phase II trials – NCT02289222 (n= 48) and NCT02036502 (n=62) – reported a very good response and acceptable safety profile for the combination of Pembrolizumab (Pem) with Lenalidomide (Len) or Pomalidomide (Pom) and Dexamethasone (Dex) in MM patients (216, 217). Another phase II trial (NCT02331368) (n=32) Pem in the early post-ASCT period was considered safe, and Pem with low Len dose was able to maintain and extend post-ASCT responses in a subgroup of patients (218). However, the immune-related adverse events and related toxicity of anti-PD-1-IMiDs combinations were unpredictable in RRMM trials, leading to patient mortality. The FDA determined that the benefit/risk ratio of the combination in RRMM is not worth the continuation of anti-PD-1-IMiDs trials.

Other combination regimens, such as anti-PD-1-radiation therapy, anti-PD-1-tumor vaccination, or combinations with other IR blockades may enhance the prognosis of refractory MM patients. Currently, blocking PD-1 with CARs has attracted the interest of investigators, with a new phase II trial (NCT04162119) recruiting patients to explore the safety and efficacy of BCMA-PD1-CAR-T cells in RRMM. BCMA-PD-1-CAR-T cell therapy works by administering T cells modified to target BCMA and secrete a PD-1Fc fusion protein capable of blocking the PD-L1/PD-1 inhibitory axis.

Given that low numbers of anti-MM T cells are a commonality amongst relapsed patients while NK cell-mediated MM cytotoxicity can be enhanced by anti-PD-1 therapy (219), the potential to harness anti-PD-1 therapy through NK cells to complement T cell-based therapies is still of interest. Therefore, a focus on combination therapies specifically enhancing the NK cell MM response may be of value.

**LAG-3** is a transmembrane protein expressed on activated T cells, NK cells, B cells, and dendritic cells (220–224). Best known for its diverse function in T cells, LAG-3 acts to control T cell activation and proliferation, while inhibiting T-reg function (221, 225). LAG-3 negatively regulates T cells by competing with CD4 in the binding of human leukocyte antigen class II (HLA-II) (226). LAG-3 can further inhibit T cell activation through binding FGL-1 independent of HLA-II. Targeting the LAG3-FGL-1 axis in murine models with mAbs promotes the antitumor T cell response (227–229)

While PD-1 and CTLA-4 were the focus of initial immune checkpoint therapies, LAG-3 is part of the next wave of IRs being clinically investigated (230). Studies demonstrated strong co-expression of PD-1 and LAG-3 on antitumor T cells (231). Combination treatment with mAbs against both negative receptors significantly reduced tumor growth in mouse models that were unresponsive to monotherapy mAb treatment, which indicates synergy between the PD-1 and LAG-3 inhibitory pathways (232). Other mouse studies revealed that anti-LAG-3 mAbs enhanced anti-PD-1 therapies and increased the secretion of activating cytokines released by tumor-infiltrating T cells (233).

In MM, one study looked at immune checkpoint expression in the pathological shift from smoldering MM to symptomatic MM and demonstrated that LAG-3 expression on T cells increased with disease progression, suggesting LAG-3 as a potential target for immunotherapy (234). Investigating biological markers on T cells after autologous stem cell transplants of MM patients, high LAG-3 expression on peripheral blood T cells post-transplant was associated with a lower event-free survival (235). This observation is supported by another study that also showed high T cell LAG-3 expression post-transplant was linked to poor prognosis of MM patients (236). The role of LAG-3 on NK cells in MM is an area of ongoing investigation.

Although LAG-3 is expressed on NK cells, it should not be considered a canonical immune checkpoint because of its low or absent expression in healthy patients (230, 237, 238). Much of the role of LAG-3 in NK cells is still unknown; however one study showed that LAG-3 expression on NK cell contributes to the effectiveness of anti-LAG-3 mAbs (238).

A more recent analysis of the status of LAG-3 on the NK cell surface following exposure to IFN-α demonstrated an increased expression of LAG3 (239). This paper proposes more studies on the impact of other cytokines on these IRs, and questions whether a single cytokine or a group of them cooperate and upregulate the IR, and/or one cytokine triggers one or array of other chemokines.

Further characterization showed that LAG3 was expressed in the NK cells populations that show high expression of activation and maturation markers. Additionally, *in-vitro* LAG3 blocking on NK cells using mAb led to an increase in the production of cytokines IFN-γ, TNF-α, MIP-1α and MIP-1β, without affecting the cytotoxic activity, which suggests that LAG3 is a negative regulator of cytokine production by mature NK cells (239).

An anti-LAG-3 mAb is currently under clinical investigation in hematological malignancies (**Table 1**).

## Perspectives

Targeting IRs with mAbs has shown preliminary success in early clinical trials with positive response rates for some cancers. Emerging evidence also suggests that targeting IRs expressed on NK cells in MM remains a viable option and requires further exploration with particular attention paid to understanding the heterogeneity in ligand expression both within and across MM patients, the interplay between NK and T cells in response to IR blockade therapy, and how NK-targeted therapy can be combined with existing therapeutic options in MM patients.

In this review, we have highlighted the preclinical evidence that IRs on the NK cell such as KIRs, NKG2A, TIGIT, TIM-3, PD-1, and LAG-3 may impact MM biology and response to treatments. KIRs remain the most promising target. Not only were anti-KIR antibodies shown to be well tolerated, but they were also shown to enhance NK cell function (133, 134). As monotherapy, a phase I clinical trials showed that targeting KIRs in monotherapy increased NK cell cytotoxicity against MM cells *ex-vivo* (NCT00552396). Another phase I clinical trial of anti-KIR in combination with lenalidomide demonstrated positive objective responses in a subset of patients (NCT01217203). Although some of the phase II trials did not report significant patient responses, we argue that a lack of understanding regarding the expression of KIRs limits our ability to predict positive responses in clinical trials.

Many of the IRs such as TIGIT, LAG-3, PD-1, and VISTA are expressed not only on NK cells, but also on T cells. Theoretically, blocking an IR expressed on both NK cells and T cells should enhance the anti-cancer effects of both immune cell types. However, the bulk of research on these IRs, particularly in the case of PD-1 and VISTA, has only elucidated their role on T cells, while neglecting to explore the role of NK cells. This is the case within the MM field as well as within the broader cancer community, highlighting the need for a more comprehensive understanding of how each immune cell type independently and collectively contributes to an anti-cancer effect. Specifically, the importance of NK cells has been shown in a study where the presence of NK cells affects the efficacy of a TIGIT-blocking mAb (181). NK cells serve as an important mediator of the immune response that have several advantages over T cells. For example, T cell activation requires both antigen recognition *via* the TCR using restricted receptors produced by gene rearrangement followed by a second activating, costimulatory signal. NK cells, on the other hand, are equipped with a repertoire of receptors to initiate activating signals that lead to NK cell-mediated cytotoxic cell lysis and cytokine production (29, 240), making them a more attractive target than T cells. The activation mechanism for NK cells relies simply on the balanced expression of ARs and IRs where the use of IR-targeted therapies can shift this balance in favour of an anti-tumour response. Additionally, as part of the innate immune system, NK cells are the first responders and are not only able to initiate an immune response much faster than T cells but are also responsible for recruiting T cells (241). By incorporating analysis of NK cell biology along with T cell assessment in both preclinical and clinical trials, the precise role of NK cells in MM development can be better understood. There may also be NK cell IRs playing a role in MM that have yet to be elucidated. Although there is little support for their role in healthy NK cells, preliminary evidence suggests VISTA and CTLA-4 IRs may affect NK function within a diseased microenvironment.

Tailoring treatment to patient-specific expression of receptors is widely adopted within the solid tumor community, but its use in MM is still relatively new. To ensure success of IR blockade therapy in MM, it is essential to estimate the patient’s expression of NK specific inhibitory ligands on malignant MM cells as well as the expression and functionality of targetable IRs on NK cells or T cells. Similarly, assessing the NK cells’ percentage, viability and functionality prior to the initiation of therapy may predict response to therapy. Previous trials proposed that the intra-tumoral level of IRs such as PD-1 on TILs were significant determinants of success for IR mAb therapies (242). However, there is not only heterogeneity in the percentage and activity of NK cells within a MM patient population (59, 65, 67), but also in the expression of specific IRs or ligands. Such differential expression can affect activity of NK cell functionality.

With this knowledge, prescription of specific IRs mAb relevant to individual expression patterns will more likely augment the immune cells to eradicate the cancer cells by hampering their evasion strategies in a precision-focused manner.

Early failure in clinical trials blocking these IRs is likely a complicated story reflecting not only intra- and inter-patient heterogeneity discussed above, but may also reflect the impaired immune landscape that is also temporally dynamic. A better understanding of how NK cell proliferation, function, and expression of receptors or their ligands changes during disease progression as well as in response to specific chemotherapeutics will improve our ability to effectively target NK cells to enhance their anti-tumor response. Specifically, studies have shown that expression of ligands such as PVR, PD-L1 can be enhanced by chemotherapy and/or IFN-γ (191, 192, 243). Similarly, NK cell contact with malignant MM cells was shown to enhance expression of PD-1 and CD94 by a process called trogocytosis (67, 214) and LAG-3 due to the exposure to the IFN-α (239). Recent studies also show a shift in NK cell populations during MM disease progression from MGUS to active MM (66, 67). Combined with knowledge regarding receptor or ligand expression, understanding these temporal dynamics will improve targeted IR therapy and the ability to treat the right patients at the right time.

Additionally, given the complex immune landscape in which NK cells reside, blocking a single NK cell IR may be insufficient in overcoming NK cell impairment in patients with severely compromised immune systems. Combination therapies further restoring immune and NK cell function may enhance NK cell IR therapies and elicit better patient outcomes. For example, a trial assessing both TIGIT and LAG-3 targeting in combination with anti-PD-1 is ongoing (NCT04150965). Similarly, IR-targeted therapy not only has implications for the intrinsic cytotoxic capabilities of NK cells, but can also be used within an ADCC and CAR-NK cell context. Considering the unique characteristic of the NK cells, which mediating ADCC, combining mAb against specific antigens expressed on myeloma cells with mAb targeting specific NK IRs according to their functional level and their cognate ligands on myeloma cells could enhance the NK cells killing. The combination of PD-1 blockade with mAbs daratumumab or elotuzumab are intriguing possibilities currently under investigation (219). Similarly, concomitantly using CAR-NK cells or genetically engineered NK cell/NK cell lines for adoptive cellular therapy with NK IRs blockade may also enhance the NK-mediated immune response and offer an interesting strategy to treat MM patients. Despite failure of some combination strategies with PD-1/PD-L1 checkpoint blockade in MM due to significant toxicity (217), the careful selection of patient-specific combination strategies may yield more promising results.

To conclude, we can say that there is a body of knowledge supporting the role of NK cells, IRs and cancer progression, although the evidence characterizing NK cells and their subpopulations in myeloma patients or the myeloma-NK cell interaction is still lacking. Therefore, we envision some key steps and factors to be considered in order to build on the foundation of myeloma-NK cell biology:

1. Profile NK cell receptors and subpopulations, NK cell activity and abundance, and NK cell function in myeloma.
2. Profile the expression of NK cell receptor cognate ligands in myeloma.
3. The immunosuppressive nature of myeloma poses a general challenge to immunotherapies in myeloma including those involving NK cells, and understanding and overcoming this challenge is critical to success.
4. Investigate the mechanisms that control specific ligands on the surface of myeloma.
5. Study ligand expression at the transcriptional and protein levels.
6. Evaluate the role of chemokines and soluble factors released in the microenvironment and if they positively or negatively mediate NK receptors and/or their ligands.
7. Explore the integration of NK cell-based therapies with traditional myeloma therapies pre-clinically to optimize clinical trial design.
8. Pursue the promise of CAR-NK cells in clinical trials.
9. *In vitro* and *in vivo* models should be used to study:

-Myeloma–NK cell interaction
-Impact of NK cell receptor targeting on the effectiveness of mAb therapy for myeloma
-Personalizing approaches to NK cell-based therapies using knowledge of NK cell function and myeloma-NK cell ligand expression heterogeneity.
-Approaches to building CAR-NK cells as myeloma therapeutics (autologous, allogeneic, “off the shelf”)

Mobilizing NKs in MM is particularly attractive due to their natural capacity to distinguish damaged cells from healthy cells, allowing them to specifically eliminate only the damaged cells. Utilizing NK-based immunotherapy in MM remains an interesting and understudied area of research. This review highlights the important role that NK IRs may play in MM. With more research, we propose the development of a patient-specific strategy that incorporates precise IR blocking that can be adjusted according to patient-specific responses and changes due to different treatments regiments. This will involve more investigation into NK cell characteristics, their related ligands and NK cells subpopulations in MM patients as well as the MM microenvironment throughout disease stages. With this understanding comes the potential for novel IR-blockade immunotherapies regimen that could improve disease control and thus increase survival outcomes.

## Author Contributions

All authors contributed to the article and approved the submitted version. HA and TR conceptualized and designed the manuscript. JW and HA designed and created the figures and generated the clinical trial table. SG critically edited the manuscript.

## Funding

This work is supported by a grant from The Canadian Institutes of Health Research (CIHR)-The New Brunswick Health Research Foundation (NBHRF) iCT SPOR grant SMC-151513, Canadian Cancer Society (CCS) 706194 and The Terry Fox Research Institute (TFRI) 1067.

## Conflict of Interest

The authors declare that the research was conducted in the absence of any commercial or financial relationships that could be construed as a potential conflict of interest.
